# Molecular Ontogeny of First-Feeding European Eel Larvae

**DOI:** 10.3389/fphys.2018.01477

**Published:** 2018-10-23

**Authors:** Sebastian N. Politis, Sune R. Sørensen, David Mazurais, Arianna Servili, Jose-Luis Zambonino-Infante, Joanna J. Miest, Catriona M. Clemmesen, Jonna Tomkiewicz, Ian A. E. Butts

**Affiliations:** ^1^National Institute of Aquatic Resources, Technical University of Denmark, Kongens Lyngby, Denmark; ^2^Billund Aquaculture Service A/S, Billund, Denmark; ^3^Marine Environmental Science Laboratory UMR 6539, Institut Français de Recherche pour l’Exploitation de la Mer, Plouzané, France; ^4^GEOMAR – Helmholtz Centre for Ocean Research, Kiel, Germany; ^5^Department of Life and Sports Sciences, University of Greenwich, Kent, United Kingdom; ^6^School of Fisheries, Aquaculture and Aquatic Sciences, Auburn University, Auburn, AL, United States

**Keywords:** *Anguilla anguilla*, ingestion, digestion, gene expression, RNA/DNA, aquaculture

## Abstract

Digestive system functionality of fish larvae relies on the onset of genetically pre-programmed and extrinsically influenced digestive functions. This study explored how algal supplementation (green-water) until 14 days post hatch (dph) and the ingestion of food [enriched rotifer (*Brachionus plicatilis*) paste] from 15 dph onward affects molecular maturation and functionality of European eel larval ingestion and digestion mechanisms. For this, we linked larval biometrics to expression of genes relating to appetite [ghrelin (*ghrl*), cholecystokinin (*cck*)], food intake [proopiomelanocortin (*pomc*)], digestion [trypsin (*try*), triglyceride lipase (*tgl*), amylase (*amyl*)], energy metabolism [ATP synthase F0 subunit 6 (*atp6*), cytochrome-*c*-oxidase 1 (*cox1*)], growth [insulin-like growth factor (*igf1*)] and thyroid metabolism [thyroid hormone receptors (*thrαA*, *thrβB*)]. Additionally, we estimated larval nutritional status *via* nucleic acid analysis during transition from endogenous and throughout the exogenous feeding stage. Results showed increased expression of *ghrl* and *cck* on 12 dph, marking the beginning of the first-feeding window, but no benefit of larviculture in green-water was observed. Moreover, expression of genes relating to protein (*try*) and lipid (*tgl*) hydrolysis revealed essential digestive processes occurring from 14 to 20 dph. On 16 dph, a molecular response to initiation of exogenous feeding was observed in the expression patterns of *pomc*, *atp6*, *cox1*, *igf1*, *thrαA* and *thrβB*. Additionally, we detected increased DNA contents, which coincided with increased RNA contents and greater body area, reflecting growth in feeding compared to non-feeding larvae. Thus, the here applied nutritional regime facilitated a short-term benefit, where feeding larvae were able to sustain growth and better condition than their non-feeding conspecifics. However, RNA:DNA ratios decreased from 12 dph onward, indicating a generally low larval nutritional condition, probably leading to the point-of-no-return and subsequent irreversible mortality due to unsuccessful utilization of exogenous feeding. In conclusion, this study molecularly identified the first-feeding window in European eel and revealed that exogenous feeding success occurs concurrently with the onset of a broad array of enzymes and hormones, which are known to regulate molecular processes in feeding physiology. This knowledge constitutes essential information to develop efficient larval feeding strategies and will hopefully provide a promising step toward sustainable aquaculture of European eel.

## Introduction

Eel (*Anguilla* spp.) is a targeted, high-value species for aquaculture in Asia and Europe. Unfortunately, eel farming is still a capture-based industry exclusively relying on wild-caught glass eels and thus the sustainability of this industry is challenged by the present critically low stock abundance of especially European (*A. anguilla*) eel (ICES WGEEL REPORT, 2017). Hence, it is urgently needed to further develop and establish captive breeding techniques and technologies for this critically endangered diadromous fish species. However, eels do not reproduce naturally in captivity due to complex hormonal control mechanisms that relate to their long migration to native oceanic spawning areas (Vidal et al., 2004). Such maturational barriers can be overcome through hormonally assisted reproduction, which led to the first reports of Japanese eel, *A. japonica* (Yamamoto and Yamauchi, 1974) and *A. anguilla* (Bezdenezhnykh et al., 1983) offspring obtained from artificially matured fish, more than 30 years ago. Since then, extensive scientific inquiry has moved the field from individual efforts of reproductive failure toward a stable production of Japanese eel offspring (Tanaka et al., 2001). Advances in Japanese eel culture have formed the baseline for eel research, leading to improved assisted reproduction protocols for European eel (Pedersen, 2004; Palstra et al., 2005; Müller et al., 2016). However, establishment of culture technology throughout the larval stage until metamorphosis is still challenged by lack of insights on the “critical" early life history stages and dietary requirements for the unique pre-leptocephalus larvae.

As such, research has been conducted to identify natural larval eel feeding resources and early hypotheses such as eel leptocephali absorbing dissolved organic carbon or feeding on larvacean and zooplankton fecal pellets have been developed (reviewed in Miller, 2009). Thereafter, a study investigating gut contents of European eel larvae caught in the Sargasso Sea, revealed that even the smallest larvae feed on a variety of planktonic organisms and that gelatinous zooplankton could be of fundamental dietary importance (Riemann et al., 2010). Subsequently, a study on both natural and laboratory-reared larvae of the Japanese eel, estimated that leptocephali most probably feed on particulate organic matter (POM) such as marine snow and discarded appendicularian houses containing bacteria, protozoans, and other biological materials (Miller et al., 2012). However, in spite of this increasing knowledge on natural larval eel feeding ecology, most insights gained have focused on non-*anguillid* species or older *anguillid* leptocephali, beyond the first-feeding stage (Miller, 2009). Thus, the natural first-feeding regimes of *Anguilla* pre-leptocephali still remain an enigma.

Similarly, increased scientific inquiry has focused on identifying potential first-feeding diets for laboratory reared eel larvae in aquaculture, where the first exogenously feeding eel larvae was reported two decades ago (Tanaka et al., 1995). Shortly after, the transition from the pre-leptocephalus to the leptocephalus stage was achieved, when Japanese eel larvae were fed a diet based on shark egg powder (Tanaka et al., 2001). Subsequent modifications of this diet led to the first laboratory reared glass eel production (Tanaka et al., 2003). However, the unstable supply of the eggs of the spiny dogfish (*Squalus acanthias*) used as a natural resource basis to develop this diet, in combination with the “vulnerable” status of this species (Fordham et al., 2016), has moved focus to more sustainable alternatives. Promising alternative diets based on fish protein hydrolysate that had been pre-digested with integral enzymes from frozen krill (Masuda et al., 2013), or hen egg yolk and exoskeleton-free (skinned) Antarctic krill (Okamura et al., 2013) have been reported, but still with lower success compared to the shark paste. Additionally, Japanese eel larvae were observed to feed on various minute zooplankton species, suggesting that rotifers (such as *Proales similis*) could be an alternative initial food source for eel larvae (Wullur et al., 2013). Unfortunately, identifying suitable feeds for larval European eel has been rather stagnant for several decades and only recently it was documented that artificially produced European eel pre-leptocephali successfully ingested a diet based on rotifers (*Brachionus plicatilis*) with or without natural chemo-attractants (Butts et al., 2016).

Now that European eel research has succeeded in producing larvae (*via* assisted reproduction), which are able to exogenously feed, the opportunity has emerged to elaborate our knowledge on the nutritional condition of individual larvae *via* nucleic acid (RNA/DNA) content analysis (Clemmesen, 1993) and to examine physiological mechanisms regulating feeding, digestion, and growth. Hormones that regulate feeding include appetite stimulators (orexigenic factors) such as ghrelin and inhibitors (anorexigenic factors) such as cholecystokinin (Volkoff et al., 2010). During early life history, fish larvae undergo major morphological and molecular changes, where shortly after mouth opening and before first-feeding, it is possible to detect an increasing availability of digestive enzymes relating to protein, lipid and carbohydrate hydrolysis, suggesting that the onset of the molecular digestive potential is genetically pre-programmed and not only influenced by the initiation of exogenous feeding (Zambonino-Infante and Cahu, 2001). Similar to other fish species, eel larvae lack a stomach, so they probably depend on pancreatic enzymes (such as trypsin, lipase and amylase) for the extracellular hydrolysis of food (Kurokawa et al., 2002; Pedersen et al., 2003). It has also been shown that a sub-optimum nutritional composition can retard the maturational process of digestive enzymes (Krogdahl and Sundby, 1999), indicating that fish larvae are not able to handle some dietary components due to unique digestive capacities (Cahu and Zambonino-Infante, 1995). However, the maturational processes of digestive capacities can be stopped or delayed, but also enhanced depending on the dietary composition (Zambonino-Infante and Cahu, 2001). Furthermore, rearing larvae in the presence of algae (green-water) can trigger digestive enzyme production (Cahu et al., 1998), which in addition to the genetically pre-programmed enzyme synthesis can induce an early maturation of hydrolytic functions (Lazo et al., 2000).

This demonstrates the necessity for determining the species-specific molecular digestive potential and timing in order to understand the distinct nutritional predisposition and the capacity for adaptation toward utilizing dietary components, which might not occur in the corresponding natural feeding regime. As such, here we reared European eel larvae with or without the presence of an algae mix (*Nannochloropsis*, *Pavlova*, and *Tetraselmis*) from 0 to 14 dph and with or without the presence of food (rotifer paste) from 15 to 24 dph. Thereafter, we measured larval biometrics, quantified individual nucleic acid (RNA/DNA) contents and followed the relative expression of genes relating to appetite [ghrelin (*ghrl*), cholecystokinin (*cck*)], food intake [proopiomelanocortin (*pomc*)], digestion [trypsin (*try*), triglyceride lipase (*tgl*), amylase (*amyl*)], energy metabolism [ATP synthase F0 subunit 6 (*atp6*), cytochrome-c-oxidase (*cox1*)], growth [insulin like growth factor (*igf1*)], and thyroid metabolism [thyroid hormone receptors (*thrαA*, *thrβB*)]. Hence, the objectives of this study were to (i) explore the development of endocrine systems regulating appetite, ingestion and digestion by targeted gene expression; (ii) molecularly define the first-feeding window; (iii) examine the potential benefit of green-water during endogenous feeding; and (iv) investigate the effect of initiating exogenous feeding on larval biometry, nutritional condition and gene expression in European eel.

## Materials and Methods

### Broodstock Maturation and Husbandry

Broodstock was kept at the EEL-HATCH facility in Hirtshals (Denmark), where females were held in 2000 L tanks and males in 500 L tanks, equipped with a closed recirculation system, under a continuous flow rate of ∼15 L min^-1^. Light conditions were held at low intensity (∼20 lux) and 12 h day/12 h night photoperiod. Acclimatization took place over 2 weeks, in order to reach a salinity of 36 psu and temperature of 20°C. At the onset of experiments, broodstock fishes were anesthetized (ethyl *p*-aminobenzoate, 20 mg L^-1^; Sigma-Aldrich Chemie, Steinheim, Germany) and tagged with a passive integrated transponder, while initial length and weight were recorded. Farmed male fish originated from a commercial eel farm (Stensgård Eel Farm A/S) in Jutland, Denmark. Here, mean (±SD) total length and body weight were 37.10 ± 2.2 cm and 97.6 ± 15.80 g, respectively (*n* = 21). Males were matured by weekly injection of human chorionic gonadotropin (hCG, Sigma-Aldrich Chemie, Steinheim, Germany; 150 IU per male). Wild-caught female broodstock were obtained in late autumn 2015 from the Lough Neagh lake in Northern Ireland and had a mean (±SD) length and weight of 76.3 ± 4.3 cm and 875.0 ± 132.8 g, respectively (*n* = 3). Female broodstock were matured *via* weekly injections of freeze-dried carp pituitary extract based on whole glands (CPE, Ducamar Spain S.L.U., Cantabria, Spain) at a dose of 18.75 mg kg^-1^ initial body weight. Final follicular maturation was induced using the maturation inducing steroid, 17α,20β-dihydroxy-4-pregnen-3-one (DHP crystalline, Sigma-Aldrich Chemie, Steinheim, Germany).

### Water Treatment and Conditioning

Three types of water treatment were applied – Artificial seawater (ASW) for gamete activation; filtered seawater (FSW) for broodstock systems and conditioned filtered seawater (CFSW) for embryo incubation and larval rearing. ASW was prepared using filtered tab water (reverse osmosis, Vertex Puratek 100 gpd RO/DI, Vertex Technologies Inc., Huntington Beach, CA, United States) adjusted to 36 psu using Sea salt (Red Sea International, Eilat, Israel; Sørensen et al., 2016a). FSW was based on seawater (pipeline from Skagerak, Denmark) and treated through a stepwise filtering process, passing through (i) a glass bead filter (AstralPool S.A. Barcelona, Spain, 0.86 m^2^ filter area, grain size 1–1.2 mm) for coarse particle removal, then through (ii) three 20″ cartridge filter in declining steps of 10, 5, and 1 μm pore size and last through (iii) a UV lamp (MR1-220PP, 220W, UltraAqua, Aalborg, Denmark). CFSW was prepared by supplying FSW to a water conditioning system, allowing maturation of the water (>3 months) following the principle presented by Vadstein et al. (1993) and Attramadal et al. (2012). Maturation was achieved by long retention time and steady level of nutrition at a low level in recirculation fitted 3 × 15 m^3^ biofilters filled with RK-bioelements (total biomedia-surface of 2.5 mill m^2^ ∼ 78.1 m^2^ per L system water) and reservoir for automatic adjustment of temperature and water conductivity. Here, salinity was adjusted to 36 psu using artificial sea salt (Blue Treasure Reef Sea salt, Qingdao Sea-Salt Aquarium Technology Co., Ltd., China).

### Gamete Production and Embryonic Rearing

Gamete production and handling followed procedures described in Butts et al. (2014), Sørensen et al. (2016b), and Benini et al. (2018). Upon mixing of gametes, ASW was used for zygote activation ensuring a salinity of 36 psu and temperature of 20°C. Early embryos were incubated in 15 L of ASW for 1 h, from where the buoyant egg layer was gently moved into new 15 L of ASW. At 2 h post fertilization (hpf), buoyant eggs were transferred to 60 L conical egg incubators and supplied with CFSW at a flow through rate of ∼350 ml min^-1^. Gentle aeration was added after ∼10 hpf while temperature was lowered to 18°C for better embryonic development (Politis et al., 2017). Light was kept at a low intensity of ∼10 lux (Politis et al., 2014) and twice a day sinking dead eggs were purged from the bottom valve of each incubator. At ∼48 hpf, aeration was stopped, and embryos hatched at ∼56 hpf.

### Study 1: Green-Water

Directly after hatch, larvae were stocked in 2 identical rearing units, (i) a recirculation system containing seawater and an algae mix (green-water) at ∼40.000 cells/ml or (ii) a recirculation system with seawater and no-algae (control). The algae mix consisted of commercially available frozen *Nannochloropsis* (2–5 μm), *Pavlova* (5–6 μm), and *Tetraselmis* (10–14 μm) species, representing different size groups (BlueBiotech Int., Germany). Each rearing unit facilitated a sump reservoir of ∼1 m^3^, from where water entered 4 × 80 L wet/dry trickle filters filled with RK-bioelements (240 m^2^ surface area 0.12 m^2^ per L) and thereafter re-entered the sump. Here, a protein skimmer (Turboflotor 5000 single 6.0, Aqua Medic Gmbh, Bissendorf, Germany) was included for removal of waste protein. Each rearing unit was attached to 3 × 250 L tanks, each representing one of the 3 experimental larval batches, where flow rates were kept at ∼10 L min^-1^ of CFSW. Initial stocking density was in the range of ∼5000 larvae per batch in a 250 L tank containing the algae mix and ∼5000 larvae of the corresponding batch in a 250 L tank with no-algae (control).

### Study 2: First-Feeding

For this study, larvae of the same experimental batches were reared (from 0 to 14 dph) as the above mentioned experimental controls in an identical recirculation system with only seawater (no-algae) at a flow rate of ∼10 L min^-1^ of CFSW. On 14 dph, ∼75 larvae (25 of each batch) were gently transferred to each of 36 acrylic 2 L flow through jars (drz400sm hank, Jug Desk Type, Taipei, Taiwan). The CFSW was again filtered (0.2 μm cartridge filter, CUNO 3M^®^, St. Paul, MN, United States) and then pumped into the bottom of each jar at a flowrate of ∼10 ml × min^-1^. All jars were randomly arranged, temperature was kept at 18°C (Politis et al., 2017, 2018), while light regime was set to 12 light/12 dark photoperiod and intensity of 21.5 ± 3.9 μmol m^-2^ s^-1^ (Butts et al., 2016). From 15 dph onward, 18 experimental jars received no-food (control), while the other 18 jars were fed an enriched rotifer (*Brachionus plicatilis*) paste diet (Butts et al., 2016) twice a day. Each portion weighed 706.5 ± 89.0 mg with a dry matter content of 41.3 ± 3.8 mg (*n* = 8). Composition of enrichment was: 5% moisture, 56% proteins and 17% lipids as well as 37mg/g DW n-3 HUFA and >5 DHA/EPA (ORI-ONE^®^; Skretting, Norway).

### Larval Biometry

Here, ∼15 larvae per batch (3×) of each treatment for study 1 (algae/control) were randomly sampled on 4, 8, 12, and 14 dph, while ∼15 larvae per replicate (3×) and treatment for study 2 (food/control) were randomly sampled on 15, 16, 18, 20, and 22 dph. Larvae were anesthetized using MS-222 (Sigma-Aldrich Chemie, Steinheim, Germany) and photographed using a zoom stereomicroscope (SMZ1270i fitted DS-Fi2 Camera Head, Nikon Corporation, Tokyo, Japan) for assessment of larval standard length, yolk-sac, and oil drop area as well as total body area using the NIS-Elements D software (Nikon Corporation, Tokyo, Japan).

### Gene Expression

For molecular analysis, ∼30 larvae from each replicate were randomly sampled at 4, 8, 12, and 14 dph in study 1 (algae/control) and at 15, 16, 17, 18, 20, and 22 dph in study 2 (food/control). Those larvae were euthanized using MS-222, rinsed with deionized water, preserved in a RNA later (Stabilization Reagent) and kept at -20°C following the procedure suggested by the supplier (Qiagen, Hilden, Germany). RNA was then extracted using the NucleoSpin^®^ RNA Kit (Macherey-Nagel, Germany) following the manufacturer’s instructions. RNA concentration (264 ± 230 ng μl^-1^) and purity (260/280 = 2.13 ± 0.03, 230/260 = 2.23 ± 0.12) were determined by spectrophotometry using Nanodrop ND-1000 (Peqlab, Germany) and normalized to a common concentration of 100 ng μl^-1^ with HPLC water. From the resulting total RNA, 680 ng were transcribed using the qScript^TM^ cDNA Synthesis Kit (Quantabio, Germany) according to the manufacturer’s instructions, including an additional gDNA wipe out step prior to transcription [PerfeCta^®^ DNase I Kit (Quantabio, Germany)].

The expression levels of 11 target and 2 reference genes were determined by quantitative real-time PCR (qRT-PCR), using specific primers. Primers were designed using primer 3 software v 0.4.0^1^ based on cDNA and predicted cDNA sequences available in Genbank databases (Table 1). All primers were designed for an amplification size ranging from 75 to 200 nucleotides. The elongation factor 1 α (*ef1α*) and 40S ribosomal S18 (*rps18*) genes were chosen as housekeeping genes since qBase+ software revealed that these mRNA levels were stable throughout analyzed samples (*M* < 0.4); M gives the gene stability and *M* < 0.5 is typical for stably expressed reference genes (Hellemans et al., 2007).

**Table 1:** Sequences of European eel (*Anguilla anguilla*) primers used for amplification of genes by qRT-PCR.

Full name	Abbreviation	Function	Database	Accession number	Primer sequence (5′ 3′) (F: Forward; R: Reverse)
Prepro-Ghrelin^∗^	*ghrl*	Appetite	GenBank Nucleotide	AZBK01848791	F: CCCACTGTGAGCTTCAGACA
					R: TGGACAGAGTCCATCCACAG
Cholecystokinin^∗^	*cck*	Appetite	GenBank Nucleotide	AZBK01795176	F: CGCCAACCACAGAATAAAGG
					R: ATTCGTATTCCTCGGCACTG
Trypsin	*try*	Digestion	GenBank Nucleotide	MH001533	F: TGCAGATCAAGCTCAGCAAG
					R: ATCGTTGGAGCTCATGGTGT
Triglyceride lipase	*tgl*	Digestion	GenBank Nucleotide	DQ493916	F: CTGACTGGGACAATGAGCGT
					R: CGTCTCGGTGTCGATGTAGG
Amylase^∗^	*amyl*	Digestion	Eel loci	g472	F: AGACCAACAGCGGTGAAATC
					R: TGCACGTTCAAGTCCAAGAG
ATP synthase F0	*atp6*	Energy metabolism	GenBank Nucleotide	NC_006531	F: GGCCTGCTCCCATACACATT
subunit 6					R: GACTGGTGTTCCTTCTGGCA
Cytochrome-	*cox1*	Energy metabolism	GenBank Nucleotide	NC_006531	F: CTACTCCTCTCCCTGCCAGT
*C*-Oxidase					R: CTTCTGGGTGGCCGAAGAAT
Proopiomelanocortin	*pomc*	Food intake	GenBank Nucleotide	JX441983	F: GCCTGTGCAAGTCTGAACTG
					R: GACACCATAGGGAGCAGGAA
Insulin like growth	*igf1*	Growth	GenBank Nucleotide	EU018410	F: TTCCTCTTAGCTGGGCTTTG
factor 1					R: AGCACCAGAGAGAGGGTGTG
Thyroid Hormone A	*thrαA*	Thyroid metabolism	GenBank Nucleotide	KY082904	F: GCAGTTCAACCTGGACGACT
Receptor α					R: CCTGGCACTTCTCGATCTTC
Thyroid Hormone	*thrβB*	Thyroid metabolism	GenBank Nucleotide	KY082907	F: GAAGACTGAGCCCTGAGGTG
Receptor β B					R: AGGTAATGCAGCGGTAATGG
Elongation	*ef1a*	Housekeeping	GenBank Nucleotide	EU407824	F: CTGAAGCCTGGTATGGTGGT
Factor 1 α					R: CATGGTGCATTTCCACAGAC
40S Ribosomal	*rps18*	Housekeeping	GenBank TSA	GBXM01005349	F: TGACCGATGATGAGGTTGAG
S18					R: GTTTGTTGTCCAGACCGTTG


*Primers were designed from cDNA or predicted cDNA (^∗^) sequences available in Genbank databases or from loci annotation in European eel (Eel loci). The European eel genome was obtained from zf-genomics (Henkel et al., 2012). The table also lists function, corresponding database and accession number of target gene sequences, as well as position of the primer on the referenced sequence.*

Expression of genes in each larval sample from two randomly selected replicates, of each treatment and larval age were analyzed in two technical replicates of each gene using the qPCR Biomark^TM^ HD system (Fluidigm) based on 96.96 dynamic arrays (GE chips) as previously described (Miest et al., 2016). In brief, a pre-amplification step was performed with a 500 nM primer pool of all primers in TaqMan-PreAmp Master Mix (Applied Biosystems) and 1.3 μL cDNA per sample for 10 min at 95°C; 14 cycles: 15 s at 95°C and 4 min at 60°C. Obtained PCR products were diluted 1:10 with low EDTA-TE buffer. The pre-amplified product was loaded onto the chip with SSofast-EvaGreen Supermix low Rox (Bio Rad) and DNA-Binding Dye Sample Loading Reagent (Fluidigm). Primers were loaded onto the chip at a concentration of 50 μM. The chip was run according to the Fluidigm 96.96 PCR protocol with a Tm of 60°C. The relative quantity of target gene transcripts was normalized and measured using the ΔΔ Ct method (Livak and Schmittgen, 2001). Coefficient of variation (CV) of technical replicates was calculated and checked to be <0.04 (Hellemans et al., 2007).

### Nucleic Acid Analysis

For this analysis, ∼10 larvae per batch (3×) from each treatment in study 1 (algae/control) were randomly sampled at 12 and 14 dph, while ∼10 larvae per replicate (3×) and treatment in study 2 (food/control) were randomly sampled on 16, 17, 18, 20, 22, and 24 dph. Larvae were immediately euthanized using MS-222 and frozen at -20°C. Thereafter, individual larvae were homogenized using 250–300 μl of sodiumdodecyl sulfate Tris buffer (Tris 0.05; NaCl 0.1M; SDS 0.01%; EDTA 0.01; pH 8) and the tissue homogenates were treated using the method described by Malzahn et al. (2003). Subsequently, fluorescence-photometric measurements using a specific nucleic acid dye [Ethidium bromide (EB), 2.5 mg⋅ml^-1^] were used to determine RNA and DNA content. In brief, total nucleic acid fluorescence was measured using an aliquot (130 μl) of each sample after adding 25 μl of EB solution. After total fluorescence was measured, RNAse (Serva Ribonuclease A, from bovine pancreas) was used to digest all RNA for 30 min at 37°C before the remaining fluorescence of the DNA was measured, allowing for RNA fluorescence to be estimated by subtracting the DNA from the total fluorescence. By using 16S and 23S ribosomal RNA standards (Boehringer Mannheim) and measuring the RNA related fluorescence, the mass of RNA was calculated from a calibration curve, while the amount of DNA was determined by applying a slope factor to the RNA standard curve for DNA being 2.2 times higher compared to the RNA concentration slope values to account for the difference in fluorescence between the two (Huwer et al., 2011).

### Statistical Analysis

All data were analyzed using SAS statistical software (version 9.1; SAS Institute Inc., Cary, NC, United States). Residuals were tested for normality using the Shapiro–Wilk test and homogeneity of variances was tested using a plot of residuals vs. fit values (PROC GLOT, SAS Institute 2003). Data were log_10_ or arcsine square-root-transformed when data deviated from normality and/or homoscedasticity (Zar, 1996). Statistical models were used to investigate effects of green-water and first-feeding on larval biometry, gene expression, and nucleic acid (RNA/DNA) content. Here, we analyzed the data using a series of repeated measures mixed-model ANOVAs (PROC MIXED; SAS Institute 2003). Models contained treatment (algae/control or food/control) and age (4–14 dph or 15–24 dph) main effects as well as the treatment × age interaction. Akaike’s (AIC) and Bayesian (BIC) information criteria were used to assess which covariance structure (compound symmetry, autoregressive order, or unstructured) was most appropriate (Littell et al., 1996). Treatment and age were considered fixed, whereas larval batch (study 1) or replicate (study 2) was considered random. Tukey’s *post hoc* analyses were used to compare least-squares means between treatments.

### Ethics Statement

All fish were handled in accordance with the European Union regulations concerning the protection of experimental animals (EU Dir 2010/63). Eel experimental protocols were approved by the Animal Experiments Inspectorate (AEI), Danish Ministry of Food, Agriculture and Fisheries (permit number: 2015-15-0201-00696). Briefly, adult eels were anesthetized using ethyl *p*-aminobenzoate (benzocaine) before tagging and handling. Larvae of European eel were anesthetized prior to handling and euthanized prior to sampling by using tricaine methanesulfonate (MS-222). All efforts were made to minimize animal handling and stress.

## Results

### Biometry

**FIGURE 1:**
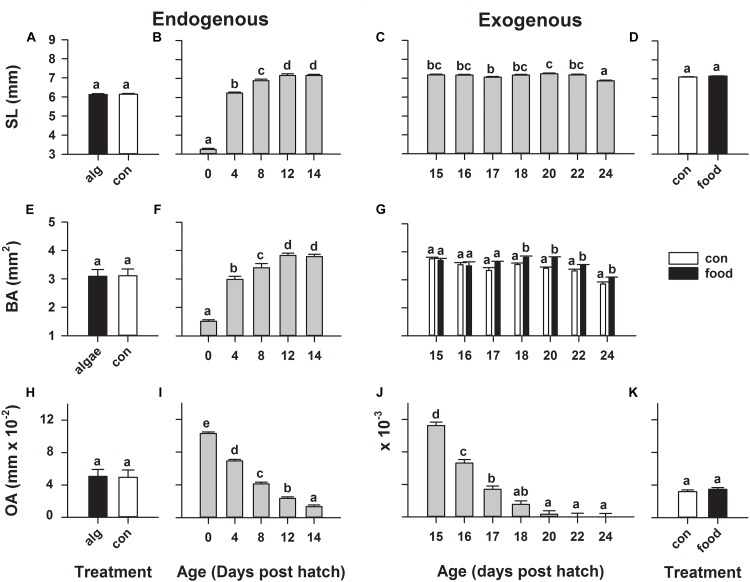
European eel (*Anguilla anguilla*) larval biometrics during endogenous feeding [algae (alg) vs. no-algae (con)] and during exogenous feeding [food vs. no-food (con)]. Standard length **(A–D)**, body area **(E–G),** and oildrop area **(H–K)**. Values represent means (±SEM) among three crosses at each age and treatment. Lower case letters represent significant differences (*p* < 0.05).

During the endogenous feeding period, from hatch until 14 dph, larvae significantly (*p* < 0.0001) grew from 3.25 ± 0.05 to 7.16 ± 0.05 mm in standard length (Figure 1B) and from 1.51 ± 0.05 to 3.79 ± 0.07 mm^2^ in body area (Figure 1F). Concurrently, larval oil drop area significantly (*p* < 0.0001) decreased from 0.103 ± 0.002 mm^2^ on 0 dph to 0.013 ± 0.002 mm^2^ on 14 dph (Figure 1I). However, rearing larvae in green-water did not have any significant influence on these larval morphometrics (Figures 1A,E,H).

During the transition to exogenous feeding, larval oil drop area further significantly (*p* < 0.0001) decreased from 0.011 ± 0.001 mm^2^ on 15 dph until it was fully utilized (Figure 1J), while larval standard length significantly (*p* < 0.0001) decreased on 24 dph (Figure 1C). However, initiation of first-feeding did not significantly alter these larval morphometrics (Figures 1D,K). Moreover, a significant (*p* < 0.0001) age × treatment (food/control) interaction was observed for larval body area, revealing that fed larvae retained a greater body area on 18, 20, 22, and 24 dph compared to non-feeding larvae; however still in a decreasing trend (Figure 1G).

### Gene Expression

**FIGURE 2:**
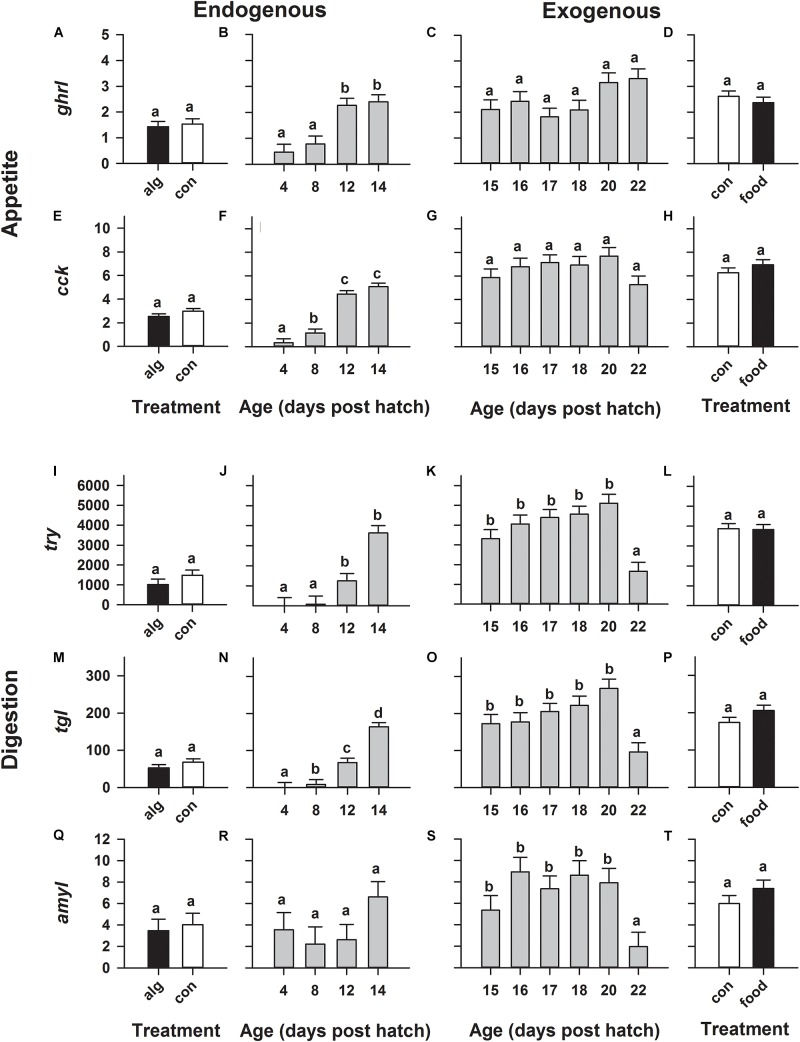
European eel (*Anguilla anguilla*) larval relative gene expression during endogenous feeding [algae (alg) vs. no-algae (con)] and during exogenous feeding [food vs. no-food (con)]. Relative expression of the appetite related orexigenic ghrelin (*ghrl*: **A–D**) and anorexigenic cholecystokinin (*cck*: **E–H**) as well as relative expression of genes encoding digestive enzymes relating to protein [trypsin (*try*): **I–L**], lipid [triclyceride lipase (*tgl*): **M–P**] and carbohydrate [amylase (*amyl*): **Q–T**] hydrolysis. Values represent means (±SEM) among three crosses at each age and treatment. Lower case letters represent significant differences (*p* < 0.05).

Expression of genes relating to appetite (*ghrl*, *cck*) significantly (*p* < 0.0001) increased during early development especially on 12 and 14 dph (Figures 2B,F), while beyond this point, their mRNA levels remained statistically constant from 15 to 22 dph (Figures 2C,G). Similarly, genes encoding digestive enzymes (*try*, *tgl*) significantly (*p* < 0.0001) increased during early development and peaked on 12–14 dph (Figures 2J,N), except for *amyl* which remained statistically constant (Figure 2R). Additionally, all digestion related genes (*try*, *tgl*, *amyl*), continued to be expressed in constant high levels (similar to the corresponding levels on 14 dph) until significantly (*p* < 0.001) dropping on 22 dph (Figures 2K,O,S).

**FIGURE 3:**
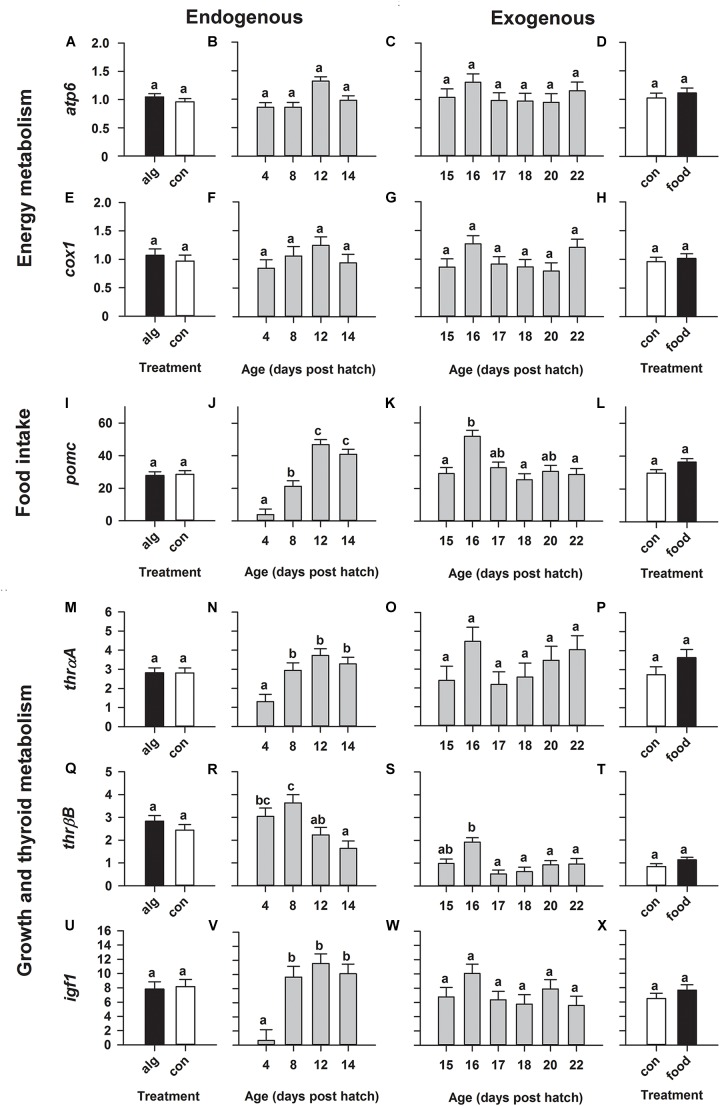
European eel (*Anguilla anguilla*) larval targeted gene expression during endogenous feeding [algae (alg) vs. no-algae (con)] and during exogenous feeding [food vs. no-food (con)]. Relative expression of genes relating to energy metabolism [ATP-synthase-F0-subunit-6 (*atp6*: **A–D**), cytochrome-*c*-oxidase (*cox1*: **E–H**)], food intake [proopiomelanocortin (*pomc*: **I–L**)], thyroid metabolism [thyroid-hormone-receptors (*thrαA*: **M–P** and *thrβB*: **Q–T**)] and growth [insulin-like-growth-factor-1 (*igf1*); **U–X**]. Values represent means (±SEM) among three crosses at each age and treatment. Lower case letters represent significant differences (*p* < 0.05).

On the contrary, expression of genes relating to energy metabolism (*atp6*, *cox1*) remained statistically constant throughout development (from 4 to 22 dph), irrespective of whether larvae were reared with or without algae as well as with or without food (Figures 3A–H). Moreover, expression relating to food intake (*pomc*) significantly (*p* < 0.0001) increased during the endogenous feeding stage with highest values on 12 and 14 dph (Figure 3J). Subsequently, *pomc* significantly (*p* = 0.006) peaked on 16 dph after the initiation of first-feeding and decreased again beyond that point (Figure 3K). Moreover, the expression of *thrβB*, relating to thyroid hormone metabolism, significantly (*p* = 0.004) decreased during the endogenous period (Figure 3R) and similarly to *pomc* showed a significant (*p* = 0.0003) peak on 16 dph following the transition to exogenous feeding; and then decreased beyond that (Figure 3S). Furthermore, expression of *thrαA* (thyroid hormone metabolism) and *igf1* (growth and development) increased during the endogenous period, reaching highest constant values already from 8 to 14 dph (Figures 3N,V) and even though they both showed an elevated (non-significant) expression on 16 dph during exogenous feeding, mRNA levels remained statistically constant from 15 to 22 dph (Figures 3O,W).

However, generally rearing larvae with algae or initiating first-feeding did not significantly alter gene expression compared to larvae reared with no algae (Figures 2A,E,I,M,Q and Figures 3A,E,I,M,Q,U) or no food (Figures 2D,H,L,P,T and Figures 3D,H,L,P,T,X), respectively.

### Nucleic Acid Analysis

**FIGURE 4:**
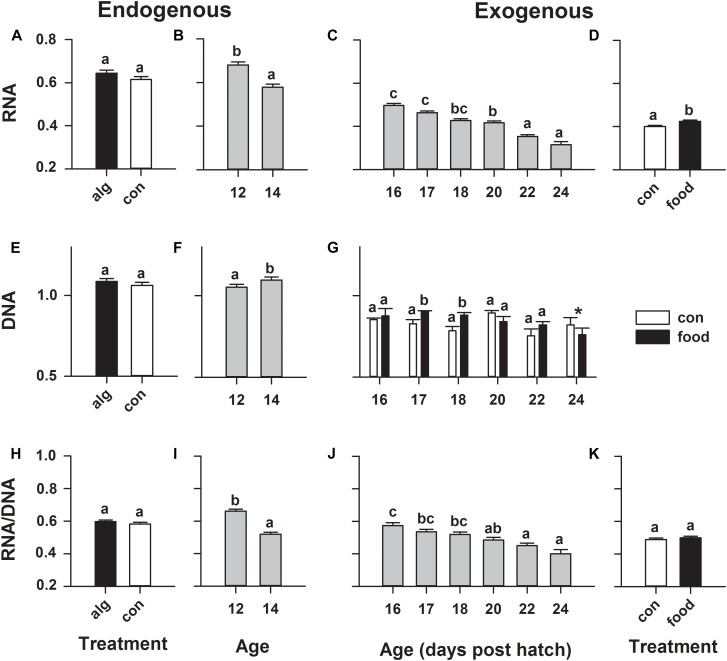
European eel (*Anguilla anguilla*) individual larval nucleic acid content during endogenous feeding [algae (alg) vs. no-algae (con)] and during exogenous feeding [food vs. no-food (con)]. Total RNA **(A–D)** or DNA **(E–G)** content and RNA:DNA ratio **(H–K)**. Values represent means (±SEM) among 5–10 individual larvae from 3 replicates at each age and treatment. Lower case letters represent significant differences (*p* < 0.05).

During endogenous feeding, the green-water principal did not significantly affect any fluorometrically measured nucleic acid content (Figures 4A,E,H), but larval age had a significant effect, where RNA content significantly (*p* < 0.001) decreased and DNA significantly (*p* = 0.015) increased, leading to a significantly (*p* = 0.001) decreased RNA/DNA ratio from 12 to 14 dph (Figures 4B,F,I). Larvae that took up first-feeding showed a constant significantly (*p* = 0.007) higher RNA content compared to non-feeding larvae (Figure 4D). A significant (*p* = 0.043) age × treatment (food/control) interaction was observed for DNA, revealing a significantly (*p* < 0.05) increased DNA content in feeding larvae on 17 and 18 dph compared to non-feeding larvae (Figure 4G). However, as both RNA and DNA were elevated in response to feeding, no change in RNA/DNA ratio was observed in response to this treatment (Figure 4K). Furthermore, it was observed that RNA and RNA/DNA ratio significantly (*p* < 0.0001) decreased throughout development (from 16 to 24 dph) irrespectively of whether the larvae were feeding or not (Figures 4C,J).

## Discussion

The nutritional requirements of fish larvae are species-specific and even differ across developmental stages within a species, mainly due to the major morphological and physiological changes during ontogeny (Holt, 2011). Comparable to most species of young marine fish (Govoni et al., 1986), the digestive system of eel larvae is undeveloped at hatch and forms into a narrow and straight digestive tract, with liver and pancreas elongated anteriorly from the middle part of the digestive tract along the esophagus, while the anus opens posteriorly (Kurokawa et al., 1995). However, the stomach differentiates only after metamorphosis into the glass eel stage, suggesting that larval eel digestion depends on the enzymatic functionality of the pancreas and gut (Kurokawa and Pedersen, 2003). During early larval digestive system development, the activity of most fish digestive enzymes is initiated before the transition from yolk-sac larvae to exogenous feeding and is thus linked to underlying genetic mechanisms (Zambonino-Infante and Cahu, 2001). Considering that genes encoding digestive enzymes were expressed irrespective of exogenous food ingestion, it seems reasonable to assume that this mechanism is linked to an internal clock, which is under endocrine control. The mechanism regulating feeding procedures includes appetite stimulators (orexigenic factors) such as *ghrl* and inhibitors (anorexigenic factors) such as *cck* (Volkoff et al., 2010). Hence, the expression of those genes (*ghrl* and *cck*) which seem to be involved in the molecular regulation of fish larval nutrition, can reveal the transition to exogenous feeding (Kurokawa et al., 2004; Ping et al., 2014). In our study, both *cck* and *ghrl* were expressed at basic levels already on 4 dph and increased toward 12 dph, indicating the molecular ontogenetic start of the first-feeding window in European eel pre-leptocephalus larvae. However, eel larvae were observed to ingest exogenous food later than the developmental functionality of the feeding apparatus (Butts et al., 2016), demonstrating the necessity for an earlier and/or improved transition to exogenous feeding. Interestingly, *cck* mRNA levels were significantly elevated prior to the first-feeding stage on 8 dph compared to the basal levels on 4 dph, indicating the potential adaptive capacity toward an earlier maturation of the digestive function and pancreatic enzyme secretion.

An early maturation of hydrolytic functions can be induced by the presence of algae during larval rearing, as shown in several fish species (reviewed in Reitan et al., 1997). In particular, it was shown that green-water during larval rearing, acts by triggering digestive enzyme production earlier than clear water in sea bass, *Dicentrarchus labrax* (Cahu et al., 1998). Nonetheless, we did not observe such a benefit of green-water during European eel pre-leptocephalus rearing. This could potentially be due to non-native algae species used in this study, not naturally occurring in the spawning area of European eel (Sargasso Sea), or the inert state of algae used which might impede triggering the desired effect of earlier maturation in digestive functionality. Moreover, improved larval growth was observed in red drum, *Sciaenops ocellatus* (Lazo et al., 2000) when live food (zooplankton) or even a microparticulate diet was supplemented with algae. In this study, the diet fed to European eel larvae was based on rotifers enriched with algae, but no further supplementation during first-feeding was tested. However, besides providing a direct nutritional supply and an indirect stimulation of appetite or digestive function, the presence of algae can influence the bacterial community of the rearing water and aid the microbial gut priming in fish larvae (Skjermo and Vadstein, 1993; Vadstein et al., 1993; Støttrup et al., 1995). Thus, it is possible that the here applied green-water rearing technique influenced the bacterial flora of the water and the microbial gut colonization, facilitating an earlier and improved larval digestion potential; however, this was outside the scope of our study. Application of supplements directing gut microbiota such as pro- and prebiotics, have received increasing attention in aquaculture, as it has been suggested that they feature a protective action on the intestinal mucosal cells, stimulating the innate immune response and thus causing an elevated state of immuno-readiness in fish such as tilapia, *Oreochromis niloticus* (Standen et al., 2013) sea bass (Franke et al., 2017), and turbot, *Scophthalmus maximus* (Miest et al., 2016). Similarly, it was shown that the dietary addition of lactic acid bacteria (probiotics) benefitted fish larvae by facilitating increased larval growth and decreased developmental deformities during early ontogeny of sea bass (Lamari et al., 2013). In this regard, the impact of algal presence or other nutritional supplementation during European eel larval rearing and the interactions with live or microparticulate diets, needs to be addressed in future research.

**FIGURE 5:**
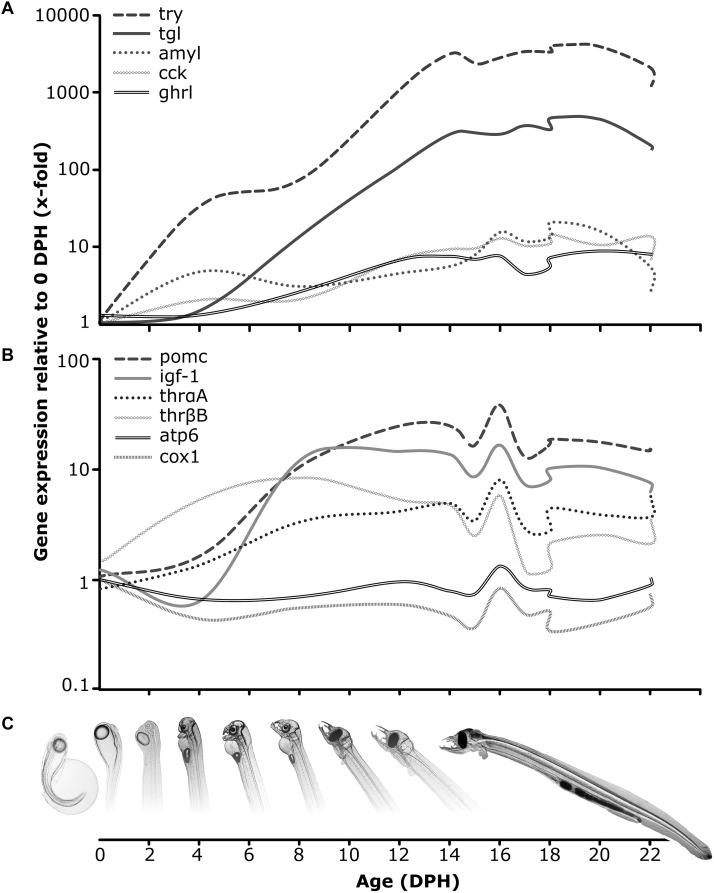
Conceptual overview – Expression (2^-ΔΔct^) was calculated in relation to the average expression on 0 days post hatch of each gene. **(A)** Relative expression for trypsin (*try*), triglyceride lipase (*tgl*), amylase (*amyl*), cholecystokinin (*cck*) and ghrelin (*ghrl*); **(B)** Relative expression for proopiomelanocortin (*pomc*), insulin-like growth factor (*igf1*), thyroid hormone receptors (*thrαA*, *thrβB*), ATP synthase F0 subunit 6 (*atp6*) and cytochrome-*c*-oxidase (*cox1*); **(C)** European eel pre-leptocephalus larval development from hatch until the feeding stage.

Gaining knowledge regarding digestive physiology during larval development for fish species, which are of interest to aquaculture, is essential for identifying adequate feeding strategies leading to improved production of healthy offspring. As such, several studies have utilized the recent advances in molecular tool availability in order to explore the molecular digestive system functionality and capacity in fish species such as Atlantic halibut, *Hippoglossus hippoglossus* (Murray et al., 2006), Atlantic cod, *Gadus morhua* (Kortner et al., 2011), catfish jundia, *Rhamdia quelen* (Silveira et al., 2013), blunt snout bream, *Megalobrama amblycephala* (Ping et al., 2014), Atlantic salmon, *Salmo salar* (Sahlmann et al., 2015), Atlantic bluefin tuna, *Thunnus thynnus* (Mazurais et al., 2015) and Senegalese sole, *Solea senegalensis* (Canada et al., 2017). Similarly, intensive scientific inquiry has been conducted to identify the molecular functionality and capacity of the digestive tract in Japanese eel larvae during the transition from endogenous to exogenous feeding, where it was demonstrated that expression levels of genes encoding the major pancreatic enzymes (such as trypsin, amylase and lipase), arise prior to or at initiation of exogenous feeding (Kurokawa et al., 2002; Pedersen et al., 2003; Murashita et al., 2013). In this study, we demonstrate the ontogeny of the digestive function in European eel larvae, *via* expression patterns of selected genes encoding some of the most important digestive enzymes relating to protein, lipid and carbohydrate hydrolysis. All enzymes were detected at basal expression levels already on 4 dph and increased throughout endogenous feeding ontogeny to reach increased values on 14 dph, corresponding to the period of increasing exogenous feeding incidences in this species (Butts et al., 2016). Moreover, we show that the transcript levels of protein (*try*) digestion enzymes were higher than those of carbohydrate (*amyl*) and lipid (*tgl*) digestion enzymes (Figure 5A), similar to the findings for pre-leptocephali and leptocephali larvae of Japanese eel (Hsu et al., 2015), indicating a nutritional predisposition for proteins during those life stages. This should also be in accordance with their natural feeding regime, as it is assumed that they feed on marine snow, primarily consisting of protein detritus (Miller et al., 2012). Similar to the Japanese eel findings (Hsu et al., 2015), we also detected elevated expression levels of *amyl* (carbohydrate hydrolysis) within the first-feeding window, which might reflect a primary mode of digestion (Zambonino-Infante and Cahu, 2001), but it cannot be considered that eel pre-leptocephalus larvae have a predisposition toward utilizing carbohydrates as a main energy source.

In this study, European eel larvae were offered a paste consisting of enriched rotifers (*Brachionus plicatilis*) as previously described (in Butts et al., 2016) and initiated feeding on 15 dph. On 16 dph, a molecular response to the initiation of exogenous feeding (Figure 5B) was observed in the expression pattern of genes relating to energy metabolism (*atp6*, *cox1*), food intake (*pomc*), growth (*igf1*) and thyroid metabolism (*thrαA*, *thrβB*). This up-regulation was not observed in non-feeding larvae and was purely driven by the expression profiles of those genes in the feeding treatments, but since it only occurred on this one time point, it was not sufficient to be detected by the applied statistical model. Nevertheless, as larvae ingested the diet resulting in similar gut fullness to previously reported findings (Butts et al., 2016), we conceive this as a temporary positive reaction to the ingestion of this exogenous diet. However, considering that this positive trend of up-regulated expression of those genes vanished already on the consecutive day, it is clear that even though larvae successfully ingested the rotifer paste diet, it apparently did not comprise the appropriate nutritional value needed to sustain growth and survival during this critical developmental stage. Concurrently, the mRNA expression of all digestive enzymes remained at an elevated level from first-feeding and throughout ontogeny until dropping on 20 or 22 dph. This, combined with the observed degeneration of larval tissue (decreased body area) and the fact that no larvae survived beyond 30 dph, indicate the end of the window of opportunity for larvae to ingest and digest exogenous food and the transition into the point-of-no-return, where larvae that failed to successfully take up exogenous feeding and assimilate ingested nutrients into growth, enter a period of irreversible starvation.

In the past years, major progress has been achieved regarding molecular tools, improving the sensitivity of analytical methods, such as the application of fluorometric techniques to investigate RNA/DNA ratios at individual level of even small organisms such as fish larvae (Clemmesen, 1993, 1994). The RNA/DNA ratio provides an indication of the protein-synthesizing potential of an organism and has been considered a valuable tool to be used as a biochemical indicator of the physiological and nutritional state as well as growth of aquatic organisms (Buckley et al., 2008; Chicaro and Chicaro, 2008). The principal of the RNA/DNA ratio is based on the assumption that under changing conditions the amount of DNA is stable within the somatic cells of a given species (and at a given developmental stage), unless the amount of cells (growth or deterioration) is changing (Foley et al., 2016). Thus, DNA content can increase throughout development, since the DNA content per cell remains constant, but the total cell number increases with growth (Ferron and Leggett, 1994). In contrast to DNA, the amount of RNA varies with changing nutritional conditions as it directly drives gene expression and protein synthesis. Thus, a recently well-fed, metabolically active, growing individual should have a relatively high RNA:DNA ratio compared to a starving, metabolically inactive individual (Buckley et al., 1999). In our study, we observed a significantly higher amount of RNA in feeding compared to non-feeding larvae, throughout the entire investigated period, which is a clear indication of an increased metabolic activity associated to protein synthesis, as a direct response to initiation of exogenous feeding. Moreover, we observed an increased amount of total DNA in feeding compared to non-feeding European eel larvae on 17 and 18 dph, which in combination with the increased amount of RNA and greater body area observed, especially on 18 and 20 dph indicates growth in feeding and faster deterioration in starved larvae.

Considering fish RNA/DNA ratios, low values are commonly correlated to starvation (Chicaro and Chicaro, 2008). European eel larval RNA/DNA ratios in our study ranged from 0.66 ± 0.01 on 12 dph to 0.40 ± 0.03 on 24 dph. Similarly low values (<0.5) have been reported for American glass eels (Laflamme et al., 2012) and only slightly elevated (0.8–1.2) for Japanese glass eels (Kawakami et al., 1999); although none of these values can be directly compared. The relatively low RNA/DNA ratios could also indicate an eel specific developmental strategy, characterized by a generally low metabolic activity during this early life phase. During this migratory phase in nature, eel offspring probably down-regulate metabolic expenses in order to survive their oceanic journey, while efficiently drifting *via* oceanic currents (Castonguay and McCleave, 1987; McCleave et al., 1998). Nevertheless, in our study, the larvae reared with the presence of algae and/or taking up first-feeding, did not show an improved RNA/DNA ratio, even if a time lag in response of a few days was taken into consideration (Peck et al., 2015). This is due to nucleic acid ratios providing a measure of growth and condition only within a recent time window (1–4 days) which depends on environmental factors such as temperature (Clemmesen, 1994; Buckley et al., 1999). Actually, the here measured RNA/DNA ratio as well as the RNA content per larva constantly decreased from 12 dph onward, indicating a low larval nutritional condition and a lack of successful nutrient assimilation. Additionally, the positive trend of greater DNA amounts in feeding larvae only lasted for a short-term period and DNA content followed the decreasing pattern of larval body area, leading to the unavoidable point of no return and subsequent irreversible starvation due to unsuccessful utilization of exogenous feeding.

To summarize, we here explored the endocrine regulation of feeding, molecularly identified the first-feeding-window and digestion potential as well as investigated the larval nutritional status and molecular response to green-water and first-feeding during the transition from endogenous to exogenous feeding in European eel larvae (Figure 5C). Thus, this study has demonstrated sensitive indicators of nutritional and molecular aspects around first-feeding. Together, this will help to better define feeding strategies during larviculture of this species, including the appropriate choice of nutrient sources, that will facilitate the digestive tract ontogeny and functionality as well as hopefully lead to improved growth and survival toward metamorphosis. In conclusion, the here applied nutritional regime facilitated a short-term benefit, where feeding European eel larvae were able to sustain growth and better condition than their non-feeding conspecifics. Even though a long-term advantage was not achieved, the knowledge gained provides a great step toward closing the life cycle in captivity and will hopefully provide a promising step toward sustainable aquaculture of this species.

## Data Availability

All relevant data is contained within the manuscript. Gene expression data are available at doi: 10.11583/DTU.7077353.

## Author Contributions

JT and IB provided funding. SP, JT, and IB designed the study. SP, SS, and IB conducted experimental work and collected samples. SP and SS analyzed morphological data and made illustrations. J-LZ-I, DM, AS, and SP selected target genes and designed primers. SP and JM conducted gene expression analysis. SP and CC conducted nucleic acid analysis. All authors contributed to data interpretation, manuscript revision, read and approved the submitted version. SP wrote original draft.

## Conflict of Interest Statement

The authors declare that the research was conducted in the absence of any commercial or financial relationships that could be construed as a potential conflict of interest.

## Footnotes

1 http://frodo.wi.mit.edu/primer3/
